# Systematic review of atopic dermatitis disease definition in studies using routinely collected health data†

**DOI:** 10.1111/bjd.16340

**Published:** 2018-04-25

**Authors:** M.P. Dizon, A.M. Yu, R.K. Singh, J. Wan, M.‐M. Chren, C. Flohr, J.I. Silverberg, D.J. Margolis, S.M. Langan, K. Abuabara

**Affiliations:** ^1^ Program for Clinical Research Department of Dermatology University of California San Francisco School of Medicine San Francisco CA U.S.A.; ^2^ Faculty of Medicine University of Ottawa Ottawa Ontario Canada; ^3^ University of California, Los Angeles David Geffen School of Medicine at UCLA Los Angeles CA U.S.A.; ^4^ Department of Dermatology University of Pennsylvania Perelman School of Medicine Philadelphia PA U.S.A.; ^5^ Department of Dermatology Vanderbilt University Medical Center Nashville TN 37204 U.S.A.; ^6^ Unit for Population‐Based Dermatology Research St John's Institute of Dermatology Guy's & St Thomas' NHS Foundation Trust and King's College London Chicago IL U.S.A.; ^7^ Departments of Dermatology, Preventive Medicine and Medical Social Sciences Northwestern University Feinberg School of Medicine Chicago IL U.S.A.; ^8^ Department of Biostatistics, Epidemiology and Informatics University of Pennsylvania Perelman School of Medicine Philadelphia PA U.S.A.; ^9^ Faculty of Epidemiology and Population Health London School of Hygiene and Tropical Medicine London U.K.

## Abstract

**Background:**

Routinely collected electronic health data obtained for administrative and clinical purposes are increasingly used to study atopic dermatitis (AD). Methods for identifying AD patients in routinely collected electronic health data differ, and it is unknown how this might affect study results.

**Objectives:**

To evaluate how patients with AD have been identified in studies using routinely collected electronic health data, to determine whether these methods were validated and to estimate how the method for identifying patients with AD affected variability in prevalence estimates.

**Methods:**

We systematically searched PubMed, Embase and Web of Science for studies using routinely collected electronic health data that reported on AD as a primary outcome. Studies of localized AD and other types of dermatitis were excluded. The protocol for this review was registered in PROSPERO (CRD42016037968).

**Results:**

In total, 59 studies met eligibility criteria. Medical diagnosis codes for inclusion and exclusion, number of occasions of a code, type of provider associated with a code and prescription data were used to identify patients with AD. Only two studies described validation of their methods and no study reported on disease severity. Prevalence estimates ranged from 0·18% to 38·33% (median 4·91%) and up to threefold variation in prevalence was introduced by differences in the method for identifying patients with AD.

**Conclusions:**

This systematic review highlights the need for clear reporting of methods for identifying patients with AD in routinely collected electronic health data to allow for meaningful interpretation and comparison of results.

## Abstract 2

**What's already known about this topic?**

- Increasingly, studies are using routinely collected data to study atopic dermatitis (AD).
- It is unclear how patients with AD are identified and whether methodological differences could have an impact on study findings.

**What does this study add?**

- We performed a systematic review of methods for identifying patients with AD in studies using routinely collected data and found differences in methods were associated with up to a threefold variation in prevalence estimates.
- We found variability in methods associated with up to a threefold variation in prevalence estimates.
- We encourage validation of methods and offer suggestions for reporting to allow for meaningful interpretation and comparison of results.

Plain language summary available online

Atopic dermatitis (AD, also known as eczema or atopic eczema) affects both children and adults, and increasing data suggest it is a systemic inflammatory disease.^1^ There is an unmet need for additional research in large, representative populations with longitudinal follow‐up and data on comorbid conditions. ‘Routinely collected’ electronic health data obtained for administrative and clinical purposes often meet these criteria and are increasingly being used to study the epidemiology, natural history and association of AD with other diseases.^2^ They could include data for clinical management (e.g. primary care databases), health system planning (e.g. health administrative data), documentation of clinical care (e.g. electronic health record data repositories) or epidemiological surveillance (e.g. cancer registries and public health reporting data). Because these data are not generated specifically for research purposes, they require careful validation to ensure accuracy and reproducibility.^2^

Unlike some conditions for which diagnosis may be based on diagnostic tests or laboratory values easily retrievable from medical records, AD diagnosis is typically based solely on clinical signs and symptoms, and physician assessment is considered the ‘gold standard’.^3^, ^4^, ^5^ Moreover, AD is clinically heterogeneous, with variable morphology, severity and clinical course, all of which can present challenges to identifying patients with AD accurately in routinely collected health data. It is possible that AD prevalence and severity estimates are influenced by the method used to identify patients. Therefore, we aimed to provide an overview of AD disease definition in studies using routinely collected data. The primary objectives of this systematic review were to evaluate how patients with AD have been identified and how disease severity was defined. We also aimed to determine whether these methods were validated (i.e. whether any information was included about the accuracy of methods for identifying AD) and, when applicable, to estimate how AD disease definitions affected the variability in AD prevalence.

## Materials and methods

The review protocol was registered in PROSPERO, (CRD42016037968, http://www.crd.york.ac.uk/PROSPERO). We followed the Preferred Reporting Items for Systematic Reviews and Meta‐Analyses (PRISMA) and Reporting of studies Conducted Using Observational Routinely collected Data (RECORD) guidelines, which are an extension of the STROBE guidelines.^2^, ^6^, ^7^

### Types of studies

We included cohort, case–control and cross‐sectional studies using routinely collected health data reporting on AD as a primary outcome. Studies that examined AD as a predictor of a separate outcome (e.g. cancer diagnosis) were not included. Routinely collected health data were defined as data collected without specific a priori research questions and developed prior to utilization for research.^2^ Data sources designed to investigate specific questions about AD or atopic diseases, such as the International Study of Asthma and Allergies in Childhood (ISAAC), birth cohorts of patients with AD, and registries of patients with AD, were excluded. Studies of localized AD, such as hand eczema or other types of dermatitis such as contact dermatitis and seborrhoeic dermatitis, were also excluded.

### Outcome

Our primary end point was the criteria used to identify patients with AD in each study. We also examined whether these criteria were validated (i.e. whether any information was included about the accuracy of methods for identifying AD), how disease severity was defined and the prevalence of AD.

### Search strategy

With the help of a professional librarian, we searched MEDLINE via PubMed, Embase and Web of Science for studies indexed until 10 April 2016. Table S1 (see Supporting Information) shows the detailed search strategy. Studies in any language were included. Because the focus of this review was to describe how AD has been defined in the mainstream published literature, we excluded literature reviews, abstracts, conference proceedings, unpublished studies, ongoing studies and the grey literature (i.e. reports and research disseminated outside of commercial publishing). We cross‐referenced review articles and reference lists to ensure completeness.

### Selection and data extraction

Three authors (M.P.D. and one of A.M.Y., R.K.S.) performed the study selection independently and in duplicate. Titles and abstracts were screened for inclusion, followed by a full‐text review if abstracts were insufficient to determine whether studies met inclusion or exclusion criteria. Discrepancies were resolved through discussion and consensus with additional authors (K.A. and S.M.L.). For each eligible study, we extracted information on the database used, country of study, study objective, patient demographics, features of algorithms used to identify individuals with AD and prevalence estimates. When possible, missing data, including specific diagnosis codes, were obtained by contacting the study author(s).

### Data synthesis and analysis

We described the characteristics of relevant studies. Features of algorithms used to identify individuals with AD were tabulated for the included studies, and the median prevalence and variability (interquartile range and ratio of 75th to 25th percentile) were calculated by subgroup. We also reported the proportion of studies in which these algorithms have been validated and described the methods of validation.

### Risk of bias

Systematic reviews often include an assessment of the risk of bias. This involves rating each included study on the methods used for selection of the study groups, comparability of groups and ascertainment of exposure and outcome.^6^, ^8^ These categorizations were not applicable to the objective of our study (i.e. we focused exclusively on how an outcome, AD, was defined), so no risk of bias assessment was performed.

## Results

### Selection and characteristics of studies

**Figure 1:**
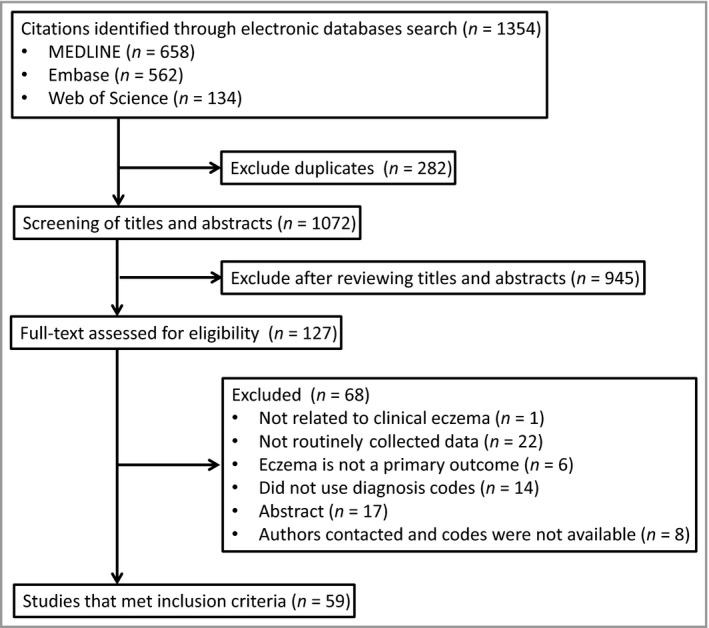
Flow diagram.

Our search identified 1354 studies. Title and abstract review identified 127 articles for the full‐text review. Of these, 68 were excluded and 59 met the inclusion criteria (Fig. 1). Table 1 shows the characteristics of the included studies. The vast majority of studies (90%) included children; only six studies (10%) included only adults. Most studies (81%) included both male and female patients, 61% were from North America or Europe, and the remainder came from East Asia. The included studies were published between 1994 and 2016, and data came from the years 1967–2014. Most studies (58%) were conducted using administrative databases (e.g. insurance databases, birth/death registries or employment registries). Primary care databases, national patient registers, institutional electronic medical records and hybrid databases compiling information from multiple sources were also used.

**Table 1:** Characteristics of the included studies

	*n* (%)
Age groups included in study sample	
Children	32 (54)
Adults	6 (10)
Both	21 (36)
Sex of patients included in study sample	
Male only	4 (7)
Female only	0
Both male and female	48 (81)
Not reported	7 (12)
Countries represented in studies^a^	
Taiwan	19
South Korea	2
U.S.A.	7
Canada	1
Sweden	5
Denmark	3
Norway	1
Finland	1
U.K.	11
The Netherlands	4
Germany	4
Australia	2
Type of routinely collected data^b^	
National patient register	3 (5)
Administrative databases (insurance, birth/death, employment)	34 (58)
Primary care databases	15 (25)
Institution‐specific electronic medical record	3 (5)
Hybrid^b^	4 (7)
Provided an estimate of atopic dermatitis prevalence	
Yes	40 (68)
No	19 (32)

^a^For some studies, data came from more than one country; ^b^hybrid datasets included patient data from sources spanning multiple categories (i.e. both primary care databases and administrative databases). National patient registers include hospital records in countries with government‐funded universal healthcare. They are not specific to insurance claims or prescriptions nor are they limited to primary care.

### Algorithm features

We categorized algorithms that were used to identify patients with AD into: (i) diagnosis codes, (ii) number of occasions of a code, (iii) type of provider associated with a code, and (iv) prescription data.

#### Diagnosis codes

Multiple codes from multiple coding systems, including International Statistical Classification of Diseases and Related Health Problems (ICD)‐9/10 and Read/OXMIS, were used. We grouped studies into those that used codes specifically for AD, and those that used codes referring to a broader group of dermatitis‐related disorders (including, for example, contact dermatitis and eczema not otherwise specified) (Table S2; see Supporting Information). When we compared the terminology used in each published study (i.e. ‘atopic dermatitis’ vs. ‘eczema’) to the types of codes, we found imperfect overlap (Table 2). Only one study directly incorporated codes for exclusionary diagnoses (‘diaper or napkin rash’ and ‘contact dermatitis and other eczema’) as part of their algorithm for AD.^9^

**Table 2:** Comparison of manuscript terminology and medical codes

Terms used in manuscript	Total	Atopic dermatitis codes only^a^	Atopic dermatitis plus other dermatitis codes^a^
Atopic dermatitis	31	26 (84)	5 (16)
Atopic dermatitis/eczema	3	0	3 (100)
Atopic eczema	5	5 (100)	0
Dermatitis/eczema	3	0	3 (100)
Eczema	17	4 (24)	13 (76)

Variables are *n* (%). ^a^A full listing of codes is detailed in Table S2 (see Supporting Information).

#### Number of occasions of a code

A total of 10 studies used algorithms that required multiple instances of codes to identify patients as having AD. Two studies^10^, ^11^ used unique algorithms that identified individuals if they had at least one inpatient claim or two outpatient claims associated with diagnosis codes for AD. Nine studies specified codes associated with AD must occur on a minimum of two occasions,^12^, ^13^, ^14^, ^15^, ^16^, ^17^, ^18^, ^19^, ^20^ and one study required three medical visits coded for AD to identify patients.^21^

#### Type of provider associated with a code

A total of seven studies, all of which were conducted in Taiwan using the National Health Insurance Research Database, specified the type of provider required to enter a diagnosis to define an individual with AD. Three studies required a diagnosis by a dermatologist,^14^, ^17^, ^22^ three studies required a diagnosis by a dermatologist or a paediatrician^15^, ^23^, ^24^ and one study required a diagnosis by a ‘specialist.’^18^

#### Prescription data

Five studies included medication prescriptions in the algorithms used to identify patients with AD. One study required a patient to have a diagnosis code and a recorded prescription of a treatment for AD (e.g. emollients, topical corticosteroids and topical calcineurin inhibitors).^25^ Two studies specified diagnosis either by diagnosis code or a prescription for calcineurin inhibitors or topical corticosteroids^26^, ^27^ and one study used only prescription codes for either topical corticosteroids or topical calcineurin inhibitors to identify patients with eczema.^28^

### Validation information

Only two studies described methods to validate the algorithms used to identify individuals with AD. The first study assessed the validity of ICD‐9‐CM codes by conducting a detailed chart review of randomly selected visits of 1000 patients.^29^ The ICD‐9‐CM diagnosis was confirmed in 93% of cases; however, the study included a range of skin diseases and was not limited to AD. The criteria used in the chart review to confirm diagnoses were not described. The second study calculated the positive predictive value of filled prescriptions of topical corticosteroids or immunosuppressants for identifying patients with an ‘umbrella diagnosis’ of dermatitis or eczema based on diagnosis codes, and found positive predictive values of 82% and 45%, respectively.^30^ The study described specific clinical criteria used to validate asthma diagnoses in a subset of paediatric patients through review of medical records; however, coded diagnoses for dermatitis and eczema were not evaluated against clinical criteria.

### Severity information

None of the studies included in the review reported on the severity of AD within the study population.

### Variation in prevalence estimates

Of the 59 included studies, 40 (68%) provided a prevalence estimate of AD, which ranged from 0·18% to 38·33%. Estimates varied by features used to identify patients with AD, study characteristics and the duration of time used to calculate the period prevalence. Of note, the variation in prevalence introduced by differences in the methods used to identify patients was similar in magnitude to the variation in prevalence introduced by study characteristics (e.g. prevalence in studies where the algorithm included prescription data was 16·9% vs. no prescription data at 4·5%; prevalence among studies including children only was 11% vs. adults and/or children at 4%, Table 3).

**Table 3:** Prevalence estimates by subgroup

	Studies, *n*	Studies with prevalence estimates, *n*	Median prevalence, %	IQR, %	Ratio of 75th to 25th percentile
Overall	59	40	4·91	2·64–11·51	4·36
Features of algorithms used to identify AD patients					
Diagnosis code category					
Limited to AD	35	26	4·30	2·14–6·70	3·13
AD plus others	23	13	11·53	9·20–30·23	3·29
Algorithm included number of visits/codes					
Required multiple occasions	8	8	4·27	3·85–5·61	1·46
No	50	31	5·60	2·24–12·37	5·52
Algorithm specified the type of provider associated with a code					
Yes	5	5	4·53	4·39–6·70	1·53
No	53	34	4·91	2·50–11·50	4·60
Algorithm included prescription data					
Yes	5	3	16·93	12·37–32·49	2·63
No	54	37	4·53	2·50–10·94	4·38
Study characteristics					
Age of patient population					
Children only	32	19	10·94	4·69–19·00	4·05
Adults +/− children	27	21	3·80	2·14–5·60	2·62
Continent					
Europe	28	21	10·35	3·80–16·93	4·46
North America	8	4	6·05	2·85–9·05	3·18
Asia	21	15	4·39	2·21–6·70	3·03
Type of routinely collected data					
National patient register	3	1	12·37	n/a	n/a
Administrative database	34	26	4·27	2·24–6·70	2·99
Primary care database	15	11	13·24	2·79–31·40	11·25
Hybrid	4	2	6·81	4·42–9·20	2·08
Terminology					
Atopic dermatitis	31	21	4·69	2·24–10·94	4·88
Atopic dermatitis/eczema	3	2	2·85	2·50–3·20	1·28
Atopic eczema	5	4	3·85	2·25–7·13	3·17
Eczema	17	13	11·50	4·42–30·23	6·84
Prevalence calculation					
Time period					
1‐year period prevalence	9	9	2·79	2·24–3·43	1·53
Multiyear period prevalence	29	29	8·90	4·15–13·21	3·18

IQR, interquartile range; AD, atopic dermatitis; n/a; not applicable.

Studies that used only diagnosis codes specific to AD to identify patients found a lower median prevalence than studies that used more general dermatitis codes (4·3% vs. 11·5%), and the amount of variability was similar (ratio of 75th to 25th percentile 3·1 vs. 3·3). Studies that required patients to have specified diagnosis codes on multiple occasions found a lower median prevalence (4·3% vs. 5·6%) and less variability (ratio of 75th to 25th percentile 1·5 vs. 5·5) than studies that only required one instance of a code. The same was true of studies that required patients to have specified diagnosis codes on multiple occasions vs. only one instance of a code found a lower median prevalence (43% vs. 56%) and less variability (ratio of 75th to 25th percentile 15 vs. 55). Finally, studies that used prescription data to identify patients found a higher median prevalence (16·9%, vs. 4·5%), versus studies that did not specify any restrictions on provider type possibly because of misclassification of patients receiving medications for other conditions.

Prevalence estimates also varied by region and age group, with the median prevalence higher in studies that included only children. The duration of period prevalence ranged from 1 to 39 years; among the nine estimates that calculated a 1‐year prevalence, the median prevalence was lower (2·8%) than in studies that calculated a multiyear period prevalence (8·9%).

## Discussion

This review demonstrates variability in the way patients with AD are identified in studies using routinely collected data. It also highlights a lack of standardization in terminology, validation studies and information on disease severity, which are all crucial to allow for comparison of study results. These issues are not specific to AD; however, they are of particular importance in AD because it is a common condition and misclassification of even a relatively small percentage of patients could result in large absolute errors.

Much has been written about the inconsistent use of terminology in allergic disorders, and efforts are under way to improve the classification system and standardize terminology used in coding.^31^, ^32^, ^33^, ^34^ To ensure we captured all relevant studies, we used multiple terms in our search, including ‘atopic dermatitis’, ‘dermatitis’, ‘eczema’ and other variants thereof (Table S1; see Supporting Information). Inconsistent use of terminology can be seen in Table 2, with some studies of ‘eczema’ including only patients with AD‐specific diagnosis codes and some studies of ‘atopic dermatitis’ including patients with diagnosis codes for other types of inflammatory skin conditions such as contact dermatitis. We found that use of broader diagnosis codes increased the median prevalence from 4·3% to 11·5% (Table 3). Such ‘lumping together’ of different disease entities could inflate AD prevalence estimates, although using more limited AD code sets might underestimate the true prevalence. For example, a recent validation study using electronic medical record data found that 42% of patients with the nonspecific diagnosis code of 692·9 and no AD‐specific code of 691·8 had a final diagnosis of AD after chart review.^35^ If a study focuses on more than one type of dermatitis, authors should clearly delineate how each condition was defined, including which codes were used. Ideally, studies examining multiple types of dermatitis would report estimates separately by subgroups to facilitate comparison with the existing literature.

This systematic review highlights the frequent use of nonvalidated algorithms to identify patients with AD in routinely collected data; only two of 59 studies described any attempt to validate the algorithms used. Validation research is a high priority to ensure patients are accurately identified and avoid misclassification bias,^36^ and since completion of our search two new validation studies of AD using routinely collected data have been published.^35^, ^37^ Both highlighted the potential magnitude of misclassification bias, even when using physician‐defined codes. For example, using a single code for ‘atopic dermatitis/eczema’, rather than one of five AD‐related codes in a primary care database from the U.K., could result in a 50% reduction in prevalence estimates.^38^

The performance of coding algorithms for identifying patients with AD is inherently context‐specific. For example, the performance of an algorithm may depend on the baseline prevalence and the way in which diagnosis codes and pharmacy codes are entered in a given setting. Moreover, in any given context, the choice of coding algorithms may be related to the goals of the study, as there is often a trade‐off between maximizing the number of true positives and reducing the number of false positives, and the value of optimizing sensitivity or specificity may depend on whether the study is aiming to identify all possible cases or to identify only those with definite disease. Therefore, each study should discuss evidence for the validity of the methods used to identify patients with AD.^2^ When possible, researchers also may consider showing how changes in their definition of AD could affect their estimates. Additional research is necessary to understand the generalizability of coding algorithms and the extent to which these might be standardized across settings.

A secondary objective of this systematic review was to examine methods used to describe AD severity in studies using routinely collected electronic health data, however, none of the studies meeting our inclusion criteria evaluated disease severity. Current approaches to measuring AD severity are often complex and not routinely documented in the medical record^39^ or are not standardized,^40^ and therefore are difficult to use in routinely collected electronic health data studies. Treatment data and/or frequency of healthcare visits has been used to define severity in studies of psoriasis and asthma using routinely collected data and may be applicable to AD.^41^, ^42^, ^43^, ^44^ Such an approach, if applied to AD, should be carefully validated.

Strengths of this systematic review include a predefined and registered protocol and adherence to reporting standards.^6^ We included 59 studies of varying designs from a variety of settings to show all of the ways patients with AD have been defined in the literature to date. We included only studies with AD as a primary outcome because we were most interested in whether differences in AD definitions would affect prevalence estimates, but were unable to synthesize these estimates using meta‐analysis because of a lack of standardized reporting of prevalence. Nonetheless, we include unadjusted median and interquartile prevalence ranges, which demonstrate variation in these estimates.

Clinicians should be aware that estimates from studies using routinely collected data may vary depending on the algorithms used to identify patients, and should be wary of studies that do not provide data on the validity of these measures. The international Harmonising Outcome Measures for Eczema (HOME) initiative was founded in response to the lack of standardization and validation of methods used to measure outcomes in randomized clinical trials, and the initiative has resulted in multiple publications suggesting standardized methods of measurement and reporting.^45^, ^46^, ^47^, ^48^, ^49^ Similar international efforts are needed for questionnaire‐based studies and studies of AD using routinely collected electronic health data. In the meantime, we encourage authors to report on their methods clearly, including: specific codes used to identify patients or exclude patients, whether there was a minimum number of codes or visits required, whether there were any restrictions on type of provider associated with the code or visit, and whether prescription data were used to identify patients. In addition, whenever possible, we encourage authors to report on the annual period prevalence of visits and/or prescriptions for AD by age to enable comparison across studies. All studies should include information on the validity of the algorithm used in their particular locale and practice setting.

## Supporting information


**Table S1** Search strategy.
**Table S2** Classification system.
**Table S3** List of included studies.

**Powerpoint S1** Journal Club Slide Set.
