# Supplementary material for: Systematic review of atopic dermatitis disease definition in studies using routinely collected health data
Source: Br J Dermatol. 2018 Apr 25;178(6):1280–7. doi: 10.1111/bjd.16340 (PMC6033033; doi:10.1111/bjd.16340)
Supplement: Supplementary file 1 — Table S1 Search strategy. Table S2 Classification system. Table S3 List of included studies. [file BJD-178-1280-s001.docx]

**Table S1**

| Classification system | |  | |  |  |
| --- | --- | --- | --- | --- | --- |
|  | ICD-9 |  |  | |  |
|  | AD |  |  | | |
|  |  | 691 | "Atopic dermatitis and related conditions" | | |
|  |  |  | "691.0" | | Diaper or napkin rash |
|  |  |  | "691.8" | | Other atopic dermatitis and related conditions |
|  | Other |  |  | | |
|  |  | 692 | "Contact dermatitis and other eczema" | | |
|  |  |  | 692.9 | | "Contact dermatitis and other eczema, unspecified cause" |
|  |  | 690 | "Erythematosquamous dermatosis" | | |
|  |  |  | 690.1 | | "Seborrheic dermatitis unspecified" |
|  |  | 373 | "Inflammation of the eyelids" | | |
|  |  |  | 373.3 | | "noninfectious dermatoses of the eyelid" |
|  |  | 705 | "Disorders of sweat glands" | | |
|  |  |  | 705.81 | | "Dyshidrosis" |
|  |  | 693 | "Dermatitis due to substances taken internally" | | |
|  |  |  | "693.0" | | "Dermatitis due to drugs and medicines taken internally" |
|  |  |  | "693.1" | | "Dermatitis due to food taken internally" |
|  |  |  | "693.8" | | "Dermatitis due to other specified substances taken internally" |
|  |  |  | "693.9" | | "Dermatitis due to unspecified substance taken internally" |
|  | ICD-10 |  |  | |  |
|  | AD |  |  | | |
|  |  | L20 | "Atopic dermatitis" | | |
|  |  |  | L20.0 | | "Besnier's prurigo" |
|  |  |  | L20.9 | | "Atopic dermatitis, unspecified" |
|  | Other |  |  | | |
|  |  | L21.9 | "Seborrheic dermatitis, unspecified" | | |
|  |  | L22 | "Diaper dermatitis" | | |
|  |  | L23 | "Allergic contact dermatitis" | | |
|  |  | L24 | "Irritant contact dermatitis" | | |
|  |  | L25 | "Unspecified contact dermatitis" | | |
|  |  |  | L25.8 | | "Unspecified contact dermatitis due to other agents" |
|  |  | L30 | "Other and unspecified dermatitis" | | |
|  |  |  | L30.8 | | "Other specified dermatitis" |
|  |  |  | L30.9 | | "Dermatitis, unspecified" |
|  | READ/OXMIS | | |  |  |
|  |  | M11 | Atopic dermatitis and related conditions | | |
|  |  | M12zz | Contact dermatitis NOS | | |
|  |  | M12z4 | Erythrodermic eczema | | |
|  |  | M12z300 | | Hand eczema | |
|  |  | M12z3 | Hand eczema | | |
|  |  | M12z200 | Infected eczema | | |
|  |  | M12z2 | Infected eczema | | |
|  |  | M12z100 | Eczema NOS | | |
|  |  | M12z1 | Eczema NOS | | |
|  |  | M12z000 | Dermatitis NOS | | |
|  |  | M12z0 | Dermatitis NOS | | |
|  |  | M12z | Contact dermatitis NOS | | |
|  |  | M128 | Allergic contact dermatitis | | |
|  |  | M11z | Atopic dermatitis NOS | | |
|  |  | M119 | Discoid eczema | | |
|  |  | M118z | Infantile seborrhoeic | | |
|  |  | M1180 | Infantile seborrhoeic dermatitis capitis | | |
|  |  | M118 | Infantile seborrhoeic dermatitis | | |
|  |  | M117 | Neurodermatitis - Atopic | | |
|  |  | M116 | Neurodermatitis - Diffuse | | |
|  |  | M115 | Besnier's prurigo | | |
|  |  | M114.00 | Allergic (intrinsic) eczema | | |
|  |  | M114 | Allergic (intrinsic) eczema | | |
|  |  | M113.00 | Flexural eczema | | |
|  |  | M113 | Flexural eczema | | |
|  |  | M112.00 | Infantile eczema | | |
|  |  | M112 | Infantile eczema | | |
|  |  | M111.00 | Atopic dermatitis/eczema | | |
|  |  | M111. | Atopic dermatitis/eczema | | |
|  |  | M111 | Atopic dermatitis/eczema | | |
|  |  | M1100 | Candidal nappy rash | | |
|  |  | M110 | Napkin dermatitis | | |
|  |  | M11.. | Atopic dermatitis and related conditions | | |
|  |  | M11. | Allergic dermatitis and related | | |
|  |  | M101.12 | Seborrheic eczema | | |
|  |  | M07z.14 | Infected dermatitis | | |
|  |  | L25 | Ingestion dermatitis | | |
|  |  | L24 | Contact dermatitis/eczema | | |
|  |  | L23 | Atopic eczema/dermatitis | | |
|  |  | L22 | Seborrhoeic dermatitis/eczema | | |
|  |  | L2.. | Dermatitis/eczemas | | |
|  |  | F5C4 | Dermatitis of eyelid | | |
|  |  | F4D31 | Contact or allergic eyelid dermatitis | | |
|  |  | 7059B | Pompholyx | |  |
|  |  | 6929PD | Perioral dermatitis | | |
|  |  | 6929NE | Eczema nummular | | |
|  |  | 6929J | Rash eczematous | | |
|  |  | 6929H | Eczema discoid | | |
|  |  | 6929EH | Eczema Hand(s) | | |
|  |  | 6929EE | Eczema Allergic | | |
|  |  | 6929CF | Eczema relapse | | |
|  |  | 6929CE | Eczema | |  |
|  |  | 6929C | Dermatitis | |  |
|  |  | 6929BE | Eczema Contact | | |
|  |  | 691EC | Eczema Atopic | | |
|  |  | 691E | Dermatitis Atopic | | |
|  |  | 691B | Infantile eczema | | |
|  |  | 691AE | Flexural eczema | | |
|  |  | 690A | Seborrheic eczema | | |
|  |  | 6869MI | Eczema infected | | |
|  |  | 6860PJ | Juvenile plantar dermatitis | | |
|  |  | 2F13 | Dry skin | |  |
|  |  | 14F1.00 | H/O Eczema | |  |
|  |  | 690 | Seborrheic dermatitis | | |

**Table S2**

Search Strategy

| PubMed – Full detail | ((("Dermatitis, Atopic"[Mesh] OR Atopic Dermatitides OR Atopic Dermatitis OR Atopic Neurodermatitides OR Atopic Neurodermatitis OR Disseminated Neurodermatitides OR Disseminated Neurodermatitis OR Atopic Eczema OR Infantile Eczema OR Eczema)))  AND  (((((("Databases as Topic"[mh] OR database*[tiab] OR "health care databases"[tiab] OR "healthcare databases"[All fields] OR "health care database"[All fields] OR "healthcare database"[tiab] OR "healthcare data"[tiab] OR "health care data"[ tiab] OR "national database"[tiab]) OR ("Registries"[mh] OR register*[tiab] OR registr*[tiab]) OR (((health information exchange[tw] OR hie[tw] OR rhio[tw] OR regional health information organization[tw] OR hl7[tw] OR health level seven[tw] OR "unified medical language system"[MeSH Major Topic] OR umls[tw] OR loinc[tw] OR rxnorm[tw] OR snomed[tw] OR icd9 cm[ti] OR icd 9 cm[ti] OR icd10[ti] OR icd 10[ti] OR metathesaurus[tw] OR patient card[tw] OR patient cards[tw] OR health card[tw] OR health cards[tw] OR electronic health data[tw] OR personal health data[tw] OR personal health record[tw] OR personal health records[tw] OR "health records, personal"[MeSH Major Topic] OR "health records, personal"[MeSH Major Topic] OR ehealth[tw] OR e-health[tw] OR "medical informatics applications"[MeSH Terms] OR "medical informatics applications"[MeSH Terms] OR "medical records systems, computerized"[MeSH Terms] OR "medical records systems, computerized"[MeSH Terms] OR computerized patient medical records[tw] OR automated medical record system[tw] OR automated medical record systems[tw] OR automated medical records system[tw] OR automated medical records systems[tw] OR computerized medical record[tw] OR computerized medical records[tw] OR computerized patient records[tw] OR computerized patient record[tw] OR computerized patient medical record[tw] OR electronic health record[tw] OR electronic health records[tw] OR "electronic health records"[MeSH Major Topic] OR "electronic health records"[MeSH Major Topic] OR electronic patient record[tw] OR electronic patient records[tw] OR electronic medical record[tw] OR electronic medical records[tw] OR electronic healthcare records[tw] OR electronic healthcare record[tw] OR electronic health care record[tw] OR electronic health care records[tw] OR "archives"[MeSH Major Topic] OR ehr[tw] OR ehrs[tw] OR phr[tw] OR phrs[tw] OR emr[tw] OR emr[tw] OR "health information systems"[MeSH Major Topic])  AND  (medical record[ti] OR "medical records"[MeSH Terms] OR medical records[ti] OR patient record[ti] OR patient records[ti] OR patient health record[ti] OR patient health records[ti] OR "patient identification systems"[MeSH Terms] OR "patient identification systems"[MeSH Terms] OR healthcare record[ti] OR healthcare records[ti] OR health care record[ti] OR health care records[ti] OR health record[ti] OR health records[ti] OR hospital information system[tw] OR hospital information systems[tw] OR umae[ti] OR "attitude to computers"[MeSH Terms] OR medical informatics[ti])) OR (("medical records systems, computerized"[MeSH Major Topic] OR "medical records systems, computerized"[MeSH Terms] OR computerized patient medical record[tw] OR computerized patient medical records[tw] OR automated medical record system[tw] OR automated medical record systems[tw] OR automated medical records system[tw] OR automated medical records systems[tw] OR computerized medical record[tw] OR computerized medical records[tw] OR computerized patient records[tw] OR computerized patient record[tw] OR electronic health record[tw] OR electronic health records[tw] OR electronic patient record[tw] OR electronic patient records[tw] OR electronic medical record[tw] OR electronic medical records[tw] OR electronic healthcare records[tw] OR electronic healthcare record[tw] OR electronic health care record[tw] OR electronic health care records[tw] OR "unified medical language system"[MeSH Major Topic] OR unified medical language system[tw] OR umls[tw] OR loinc[tw] OR rxnorm[tw] OR snomed[tw] OR icd9 cm[ti] OR icd 9 cm[ti] OR icd10[ti] OR icd 10[ti] OR Metathesaurus[tw] OR ehr[tw] OR ehrs[tw] OR phr[tw] OR phrs[tw] OR emr[tw] OR emrs[tw] OR meaningful use[tiab] OR meaningful use[tw] OR "meaningful use"[MeSH Major Topic])  AND  ("J AHIMA"[Journal] OR "J Am Med Inform Assoc"[Journal] OR "AMIA Annu Symp Proc"[Journal] OR "Health Data Manag"[Journal] OR "Int J Med Inform"[Journal] OR "Yearb Med Inform"[Journal] OR "Telemed J E Health"[Journal] OR "Stud Health Technol Inform"[Journal]))) OR (Administrative[tiab] OR Claims[tiab] OR “routine data”[tiab] OR “routinely collected”[tiab]))) NOT (animals NOT humans))) NOT ((Review[ptyp] OR Addresses[ptyp] OR Biography[ptyp] OR Bibliography[ptyp] OR Autobiography[ptyp] OR Case Reports[ptyp] OR Clinical Conference[ptyp] OR Comment[ptyp] OR Congresses[ptyp] OR Consensus Development Conference[ptyp] OR Consensus Development Conference, NIH[ptyp] OR Editorial[ptyp] OR Letter[ptyp] OR Dictionary[ptyp] OR Directory[ptyp] OR Historical Article[ptyp] OR “in vitro”[pt] OR Legal Cases[ptyp] OR Meta-Analysis[ptyp] OR Guideline[ptyp] OR News[ptyp] OR Newspaper Article[ptyp] OR Patient Education Handout[ptyp] OR Personal Narratives[ptyp] OR Practice Guideline[ptyp] OR Interview[ptyp] OR In Vitro[ptyp] OR Legislation[ptyp] OR Lectures[ptyp] OR Video-Audio Media[ptyp] OR Webcasts[ptyp] OR Portraits[ptyp])))) |
| --- | --- |
| EMBASE – Search terms | **'atopic dermatitis'**/exp OR **'atopic dermatitis'** OR **'eczema'**/exp OR **'eczema'** OR **atopic** AND **dermatitides**:ab,ti OR **atopic** AND **dermatitis**:ab,ti OR **atopic** AND **neurodermatitides**:ab,ti OR **atopic** AND **neurodermatitis**:ab,ti OR **disseminated** AND **neurodermatitides**:ab,ti OR **disseminated** AND **neurodermatitis**:ab,ti OR **atopic** AND **eczema**:ab,ti OR **infantile** AND **eczema**:ab,ti OR **eczema**:ab,ti |
| Web of Science – Search terms | (“Atopic dermatitis” OR atopic dermatitides” OR “atopic dermatitis” OR “atopic neurodermatitides” OR “atopic neurodermatitis” OR “disseminated neurodermatitides” OR “disseminated neurodermatitis” OR “atopic eczema” OR “infantile eczema” OR eczema) |

**Table S3**

| Author | Year | PMID | Title | |  |
| --- | --- | --- | --- | --- | --- |
| Anandan | 2009 | 19797601 | | Epidemiology and disease burden from allergic disease in Scotland: Analyses of national databases | |
| Augustin | 2015 | 25966818 | | Epidemiology and Comorbidity in Children with Psoriasis and Atopic Eczema | |
| Bråbäck | 2004 | 14720260 | | Trends in asthma, allergic rhinitis and eczema among Swedish conscripts from farming and non-farming environments. A nationwide study over three decades. | |
| Carey | 2003 | 14516473 | | Implications of the problem orientated medical record (POMR) for research using electronic GP databases: a comparison of the doctors independent network database (DIN) and the general practice research database (GPRD) | |
| Chang | 2005 | 15667234 | | Health plan budget impact analysis for pimecrolimus | |
| Chang | 2013 | 24278271 | | Close Correlation between Season of Birth and the Prevalence of Bronchial Asthma in a Taiwanese Population | |
| Chen | 2009 | 19171465 | | Prevalence and risk of atopic disorders among schizophrenia patients: a nationwide population based study | |
| Chen | 2013 | 23140273 | | Attention deficit hyperactivity disorder, tic disorder, and allergy: is there a link? A nationwide population-based study | |
| Chen | 2013 | 23400216 | | Comorbidity of allergic and autoimmune diseases among patients with ADHD: A nationwide population-based study | |
| Chen | 2015 | 25510261 | | Comorbidity profiles in association with vitiligo: a nationwide population-based study in Taiwan | |
| Chu | 2011 | 21616562 | | Comorbidity profiles among patients with alopecia areata: The importance of onset age, a nationwide population-based study | |
| Chung | 2012 | 22240253 | | Association of erectile dysfunction with atopic dermatitis: A population-based case-control study | |
| Egeberg | 2016 | 26950896 | | Neonatal risk factors of atopic dermatitis in Denmark - Results from a nation wide register-based study | |
| Ellis | 2002 | 11862170 | | Cost of atopic dermatitis and eczema in the United States | |
| Fleischer | 1994 | 8288916 | | Office-based physician services provided by dermatologists in the United States in 1990 | |
| Gaitatzis | 2004 | 15571520 | | The epidemiology of the comorbidity of epilepsy in the general population | |
| Grimmer | 2012 | 22031593 | | Hemangioma Is Associated with Atopic Disease | |
| Haataja | 2016 | 26898703 | | Asthma and atopic dermatitis in children born moderately and late preterm | |
| Henderson | 2012 | 22639933 | | Skin-of-Color Epidemiology: A Report of the Most Common Skin Conditions by Race | |
| Henriksen | 2015 | 25828267 | | Incidence rates of atopic dermatitis, asthma, and allergic rhinoconjunctivitis in Danish and Swedish children | |
| Hirsch | 2015 | 26332371 | | Five-year risk of incident disease following a diagnosis of chronic rhinosinusitis | |
| Hooiveld | 2016 | 26888643 | | Doctor-diagnosed health problems in a region with a high density of concentrated animal feeding operations: a cross-sectional study | |
| Horii | 2007 | 17766497 | | Atopic dermatitis in children in the United States, 1997-2004: visit trends, patient and provider characteristics, and prescribing patterns | |
| Hua | 2014 | 23980909 | | The natural course of early-onset atopic dermatitis in Taiwan: a population-based cohort study | |
| Hwang | 2010 | 21057741 | | Prevalence of atopic dermatitis, allergic rhinitis, and asthma in Taiwan: A national study 2000 to 2007 | |
| Hwang | 2013 | 23647775 | | Atopic diathesis in patients with Kawasaki disease | |
| Kaerlev | 2014 | 25411726 | | Surveillance of hospital contacts among Danish seafarers and fishermen with focus on skin and infectious diseases-a population-based cohort study | |
| Kim | 2016 | 26666496 | | Allergies are still on the rise? A 6-year nationwide population-based study in Korea | |
| Lai-Kwon | 2014 | 24359576 | | Which patients with dermatological conditions are admitted via the emergency department? | |
| Lai-Kwon | 2014 | 24800080 | | Which dermatological conditions present to an emergency department in australia? | |
| Landgren | 2006 | 16433805 | | Psoriasis in Swedish conscripts: Time trend and association with T-helper 2-mediated disorders | |
| Lin | 2016 | 26370535 | | Childhood type 1 diabetes may increase the risk of atopic dermatitis | |
| Martel | 2009 | 19616789 | | Maternal asthma, its control and severity in pregnancy, and the incidence of atopic dermatitis and allergic rhinitis in the offspring | |
| McKeever | 2001 | 11562513 | | Siblings, multiple births, and the incidence of allergic disease: A birth cohort study using the West Midlands general practice research database | |
| McKeever | 2002 | 11799364 | | Early exposure to infections and antibiotics and the incidence of allergic disease: A birth cohort study with the West Midlands General Practice Research Database | |
| McKeever | 2002 | 11994703 | | Mode of delivery and risk of developing allergic disease | |
| McKeever | 2002 | 12231492 | | The importance of prenatal exposures on the development of allergic disease: A birth cohort study using the West Midlands General Practice Database | |
| McKeever | 2004 | 15249303 | | Vaccination and allergic disease: A birth cohort study | |
| Meding | 2016 | 26280897 | | Disability pensions due to skin diseases: A cohort study in Swedish construction workers | |
| Mohammedamin | 2006 | 16551358 | | Increasing incidence of skin disorders in children? A comparison between 1987 and 2001 | |
| Mulder | 2014 | 24164287 | | Prenatal exposure to acid-suppressive drugs and the risk of allergic diseases in the offspring: a cohort study | |
| Mulder | 2016 | 26450360 | | Identification of Dutch children diagnosed with atopic diseases using prescription data: a validation study | |
| Ortqvist | 2013 | 23754713 | | Validation of asthma and eczema in population-based Swedish drug and patient registers | |
| Schmitt | 2010 | 19725897 | | Early exposure to antibiotics and infections and the incidence of atopic eczema: A population-based cohort study | |
| Shyu | 2012 | 22580087 | | Prevalence of attention-deficit/hyperactivity disorder in patients with pediatric allergic disorders: a nationwide, population-based study | |
| Simpson | 2002 | 12002734 | | Coincidence of immune-mediated diseases driven by Th1 and Th2 subsets suggests a common aetiology. A population-based study using computerized general practice data | |
| Simpson | 2008 | 19029357 | | Incidence and prevalence of multiple allergic disorders recorded in a national primary care database | |
| Simpson | 2009 | 19297652 | | Trends in the epidemiology and prescribing of medication for eczema in England | |
| Sun | 2012 | 22370527 | | Coexistence of allergic diseases: Patterns and frequencies | |
| Tien | 2014 | 24586942 | | Obstructive sleep apnea and the risk of atopic dermatitis: a population-based case control study | |
| Trønnes | 2013 | 24298940 | | The association of preterm birth with severe asthma and atopic dermatitis; a national cohort study | |
| Tsai | 2013 | 23375343 | | Association between atopic diseases and attention-deficit/hyperactivity disorder in childhood: a population-based case-control study | |
| Tsai | 2013 | 23998245 | | The association between Kawasaki disease and allergic diseases, from infancy to school age | |
| Wang | 2013 | 24062298 | | Acetaminophen and/or antibiotic use in early life and the development of childhood allergic diseases | |
| Wei | 2015 | 25843432 | | Occurence of common allergic diseases in children with idiopathic nephrotic syndrome | |
| Woon | 2013 | 24069052 | | Increased risk of atopic dermatitis in preschool children with kawasaki disease: a population-based study in Taiwan | |
| Yu | 2012 | 22690101 | | Prevalence of atopic dermatitis in Korea: Analysis by using national statistics | |
