# Supplementary material for: Systematic review of atopic dermatitis disease definition in studies using routinely collected health data
Source: Br J Dermatol. 2018 Apr 25;178(6):1280–7. doi: 10.1111/bjd.16340 (PMC6033033; doi:10.1111/bjd.16340)
Supplement: Supplementary file 2 — Powerpoint S1 Journal Club Slide Set. [file BJD-178-1280-s002.pptx]

## Slide 1
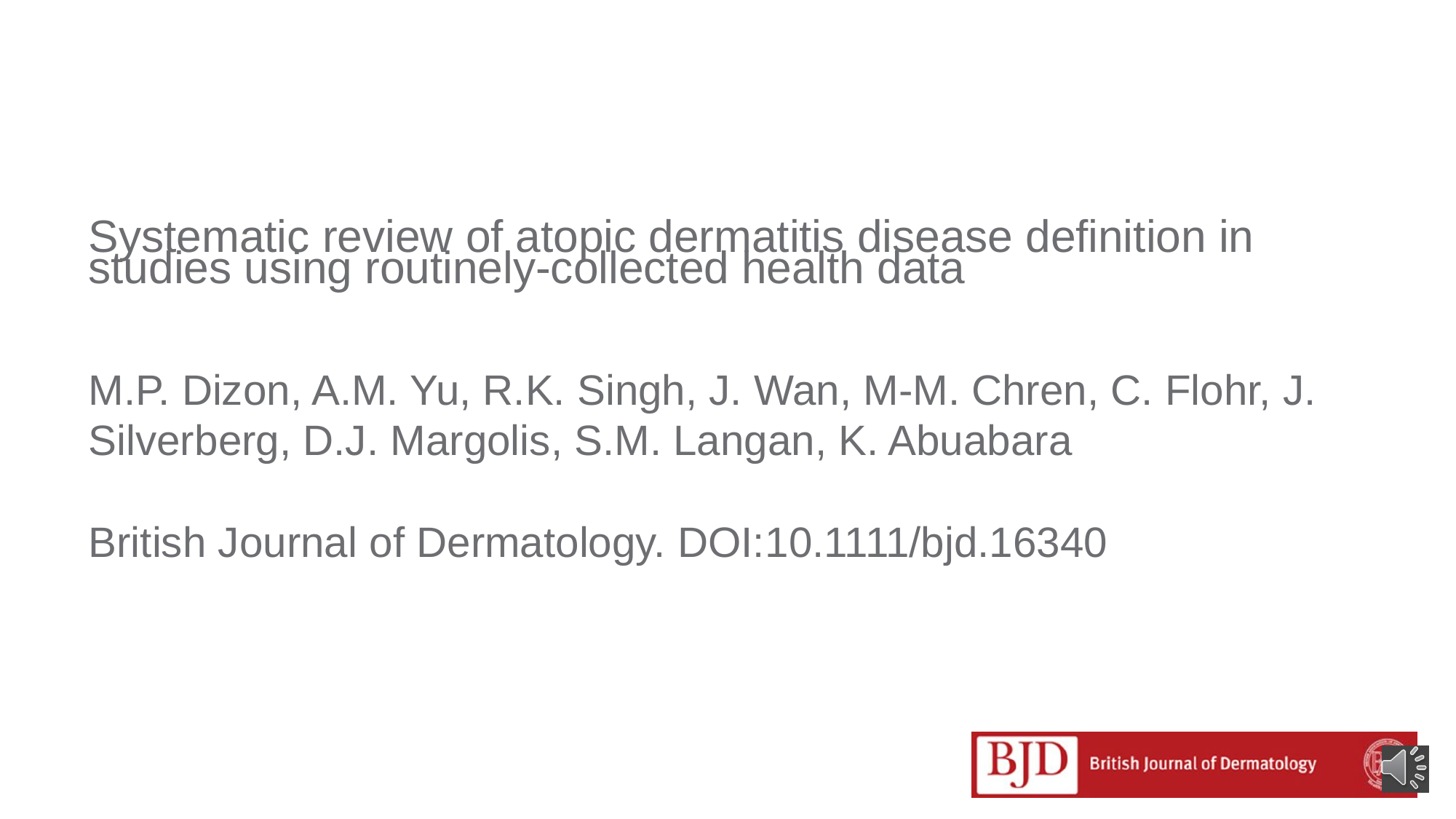

# Systematic review of atopic dermatitis disease definition in studies using routinely-collected health data
M.P. Dizon, A.M. Yu, R.K. Singh, J. Wan, M-M. Chren, C. Flohr, J. Silverberg, D.J. Margolis, S.M. Langan, K. Abuabara
British Journal of Dermatology. DOI:10.1111/bjd.16340

## Slide 2
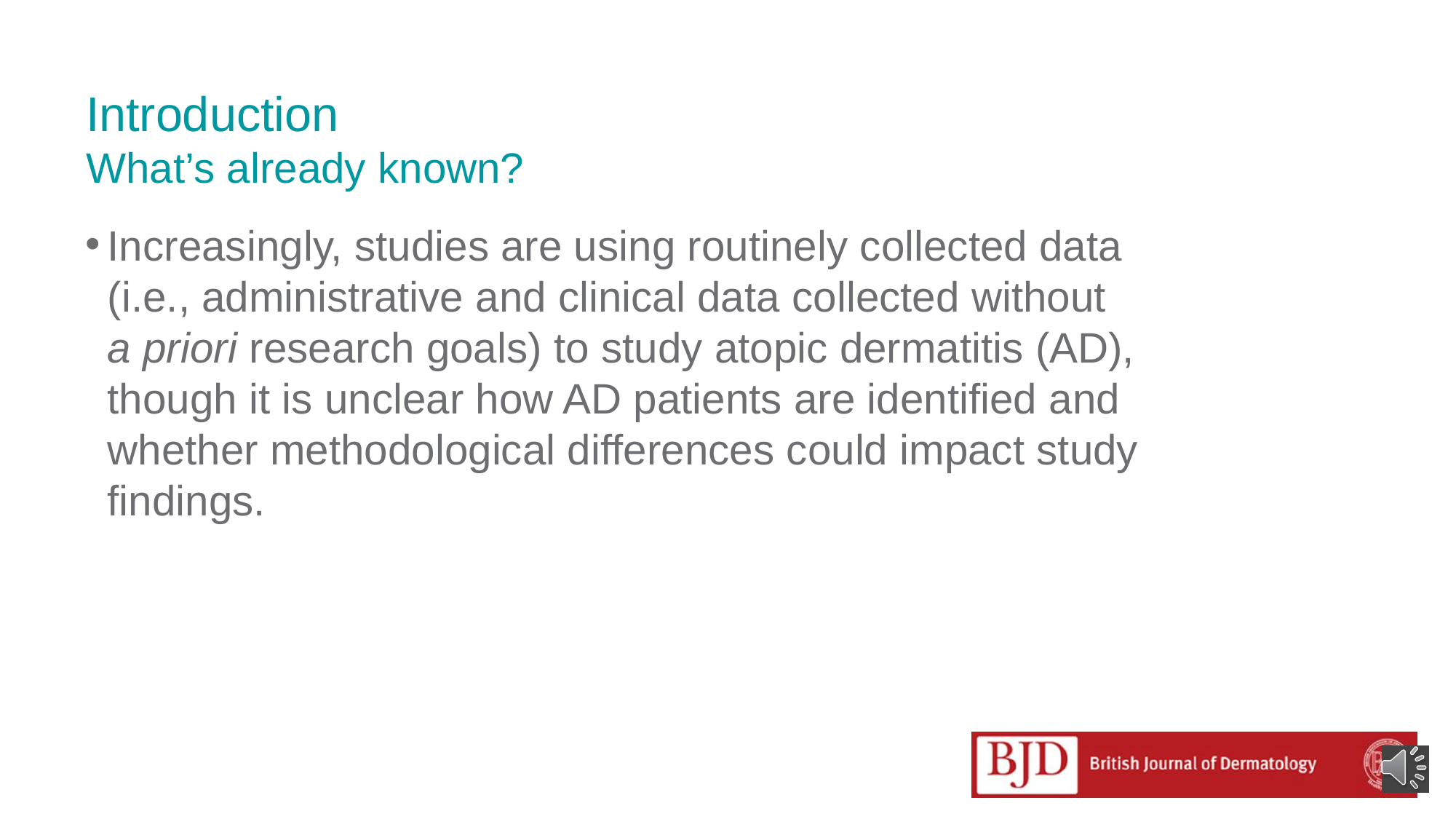

# Introduction What’s already known?
Increasingly, studies are using routinely collected data (i.e., administrative and clinical data collected without a priori research goals) to study atopic dermatitis (AD), though it is unclear how AD patients are identified and whether methodological differences could impact study findings.

## Slide 3
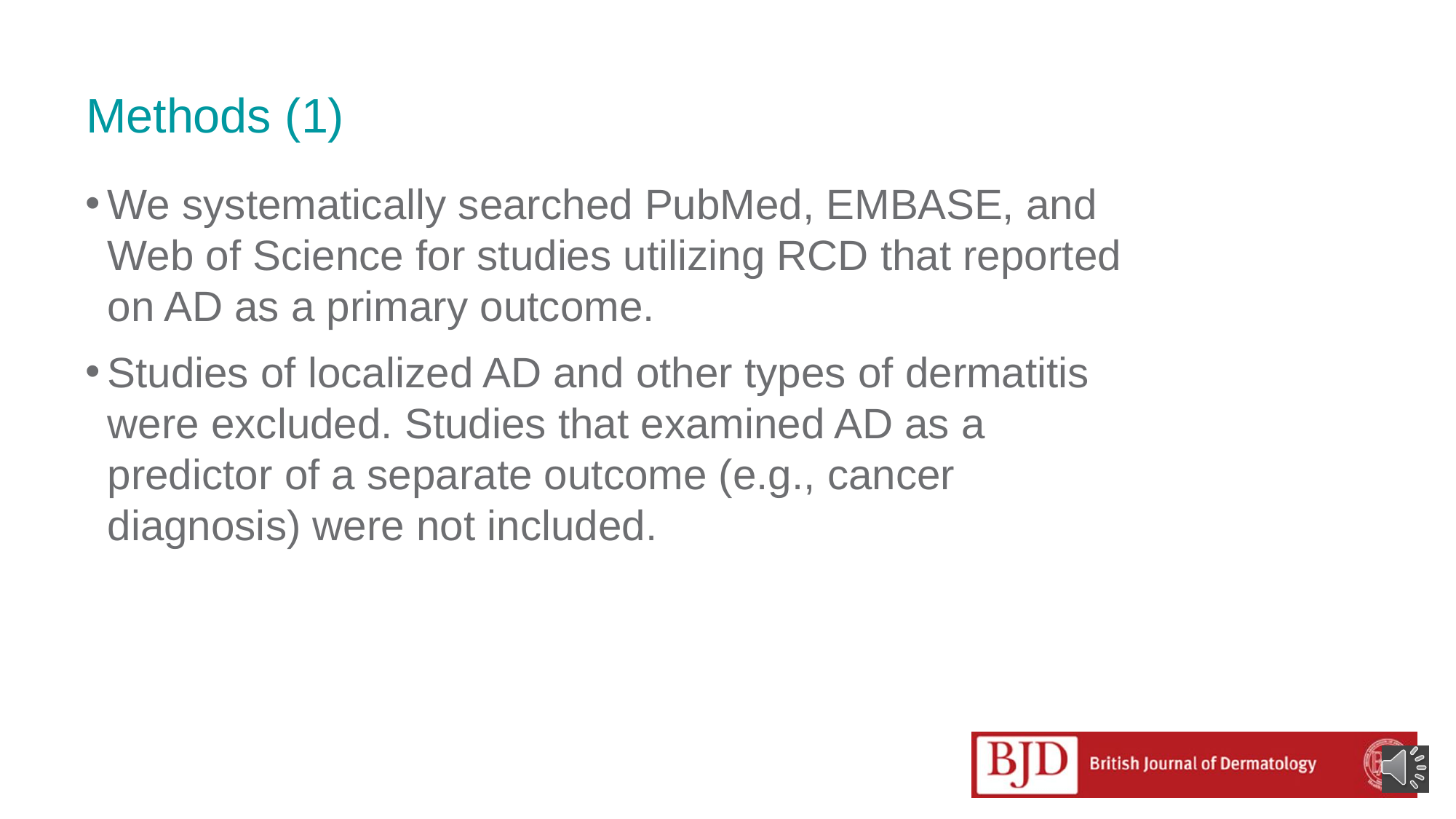

# Methods (1)
We systematically searched PubMed, EMBASE, and Web of Science for studies utilizing RCD that reported on AD as a primary outcome.
Studies of localized AD and other types of dermatitis were excluded. Studies that examined AD as a predictor of a separate outcome (e.g., cancer diagnosis) were not included.

## Slide 4
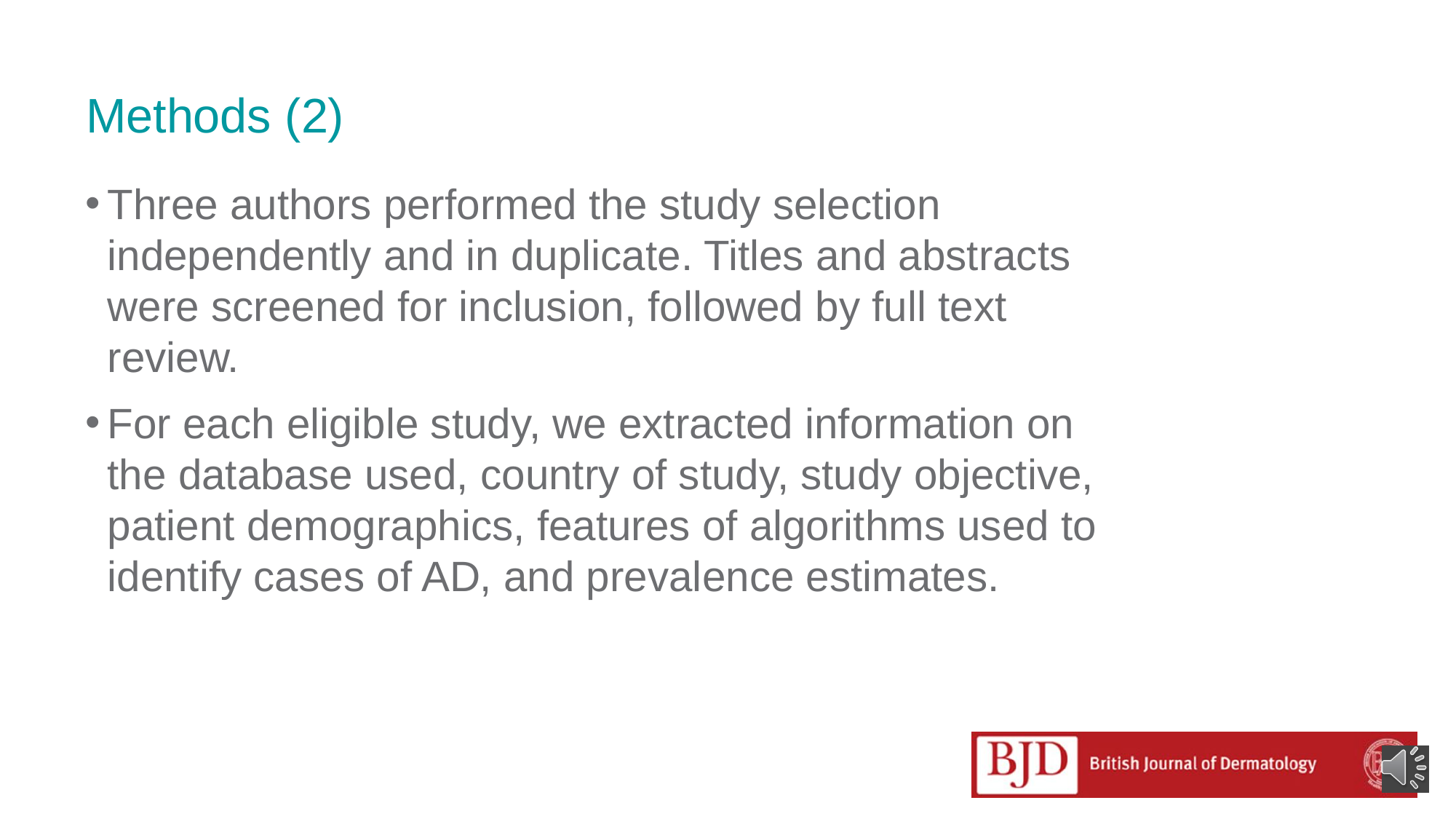

# Methods (2)
Three authors performed the study selection independently and in duplicate. Titles and abstracts were screened for inclusion, followed by full text review.
For each eligible study, we extracted information on the database used, country of study, study objective, patient demographics, features of algorithms used to identify cases of AD, and prevalence estimates.

## Slide 5
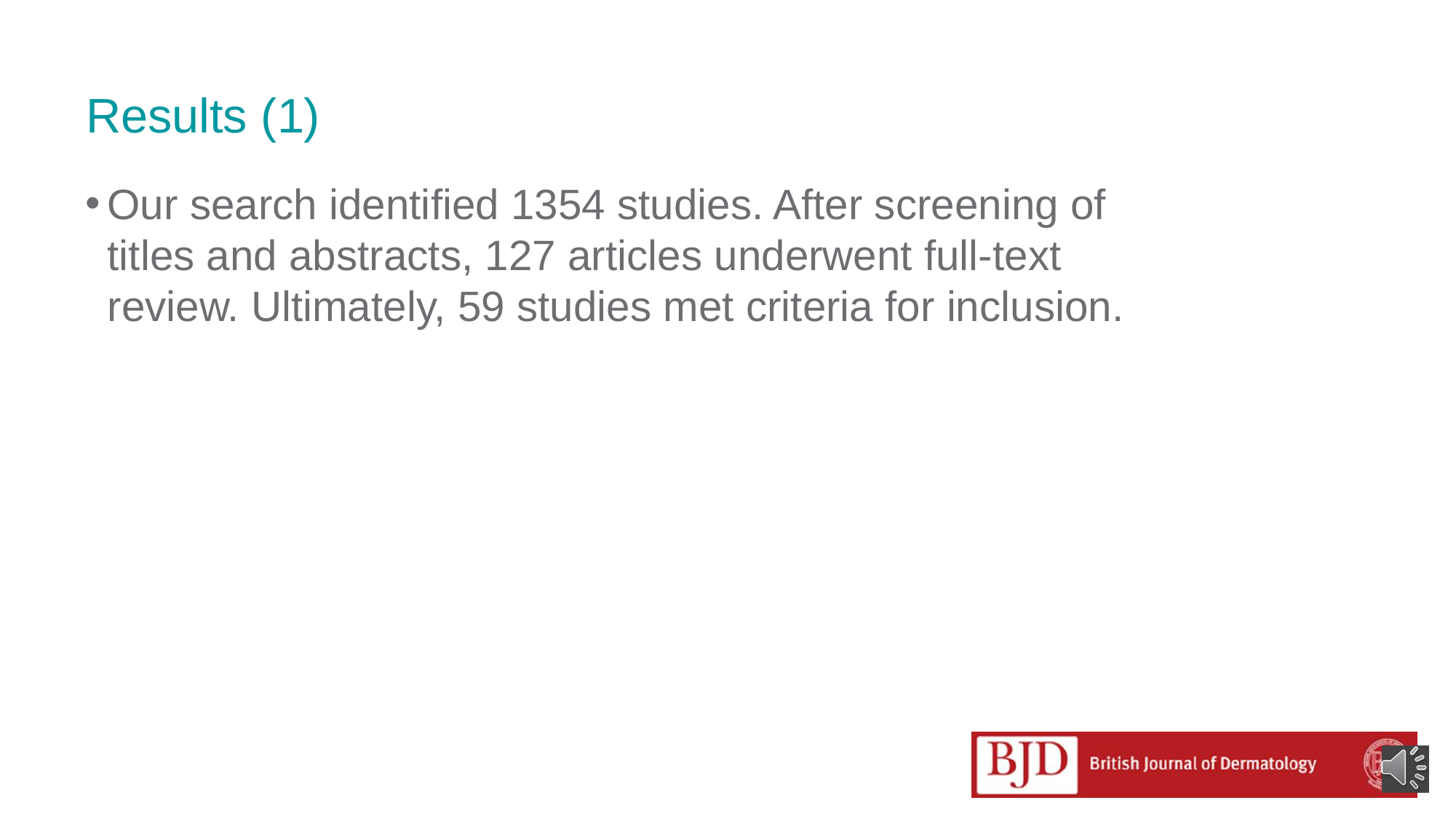

# Results (1)
Our search identified 1354 studies. After screening of titles and abstracts, 127 articles underwent full-text review. Ultimately, 59 studies met criteria for inclusion.

## Slide 6
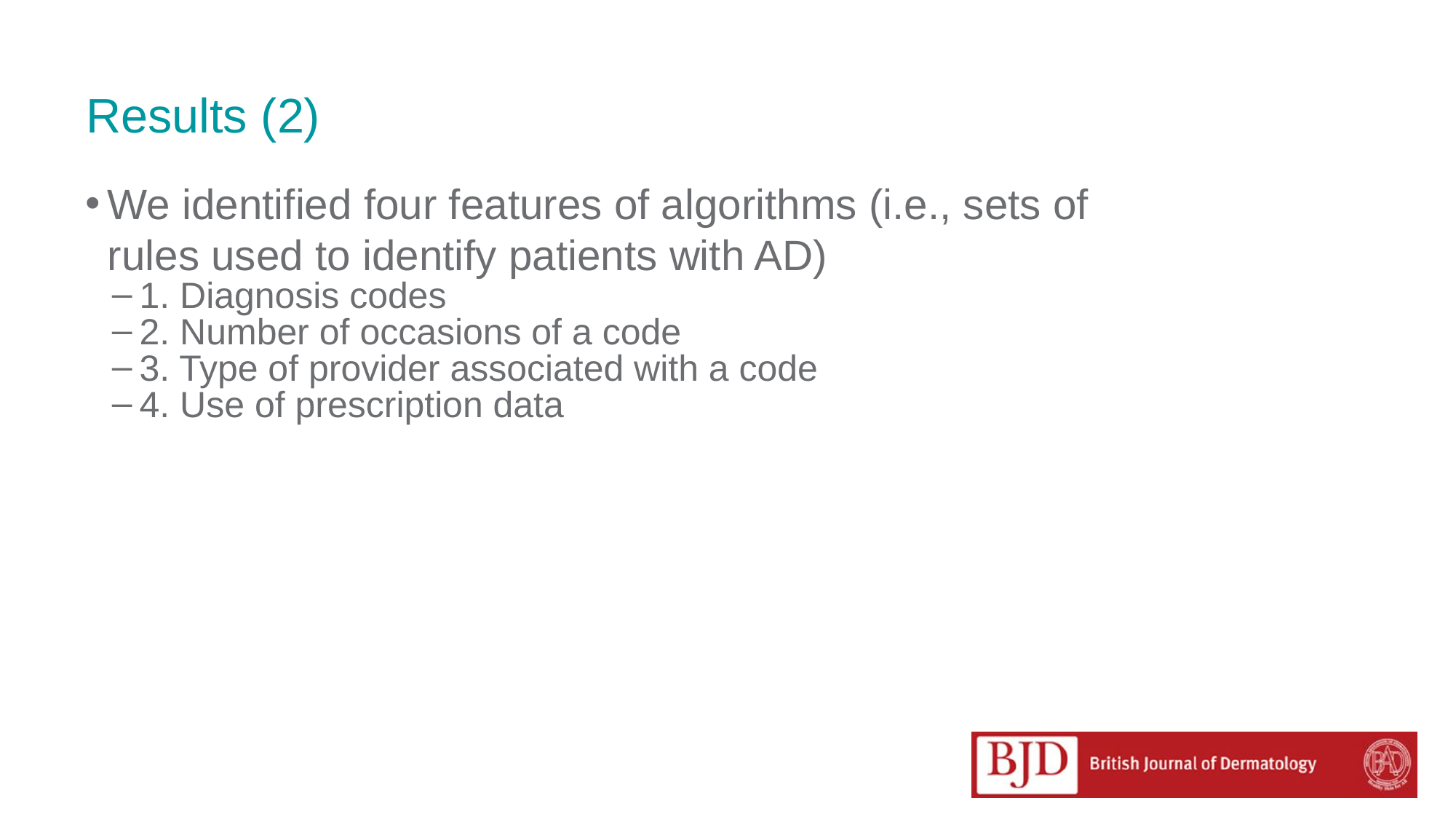

# Results (2)
We identified four features of algorithms (i.e., sets of rules used to identify patients with AD)
1. Diagnosis codes
2. Number of occasions of a code
3. Type of provider associated with a code
4. Use of prescription data

## Slide 7
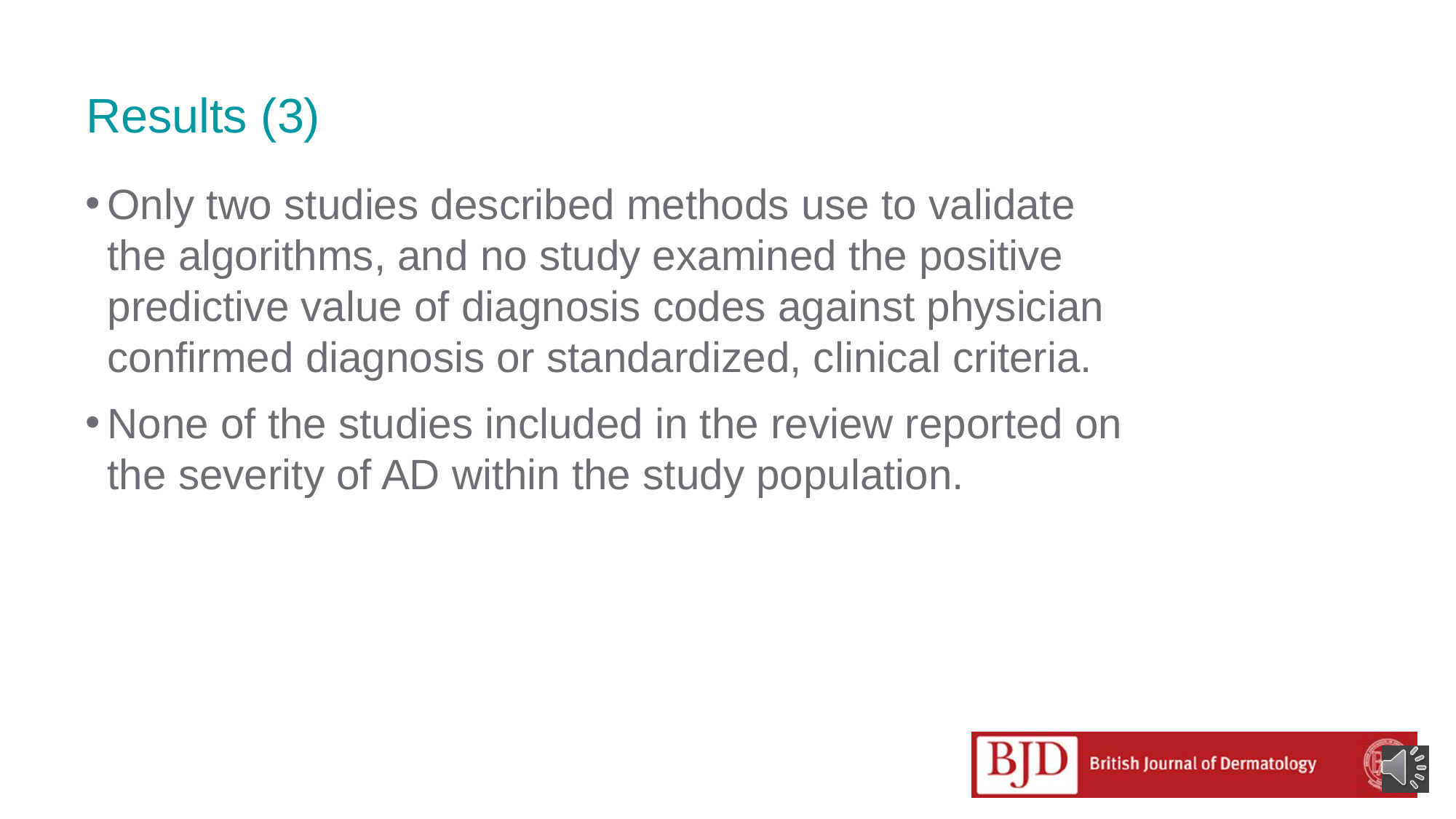

# Results (3)
Only two studies described methods use to validate the algorithms, and no study examined the positive predictive value of diagnosis codes against physician confirmed diagnosis or standardized, clinical criteria.
None of the studies included in the review reported on the severity of AD within the study population.

## Slide 8
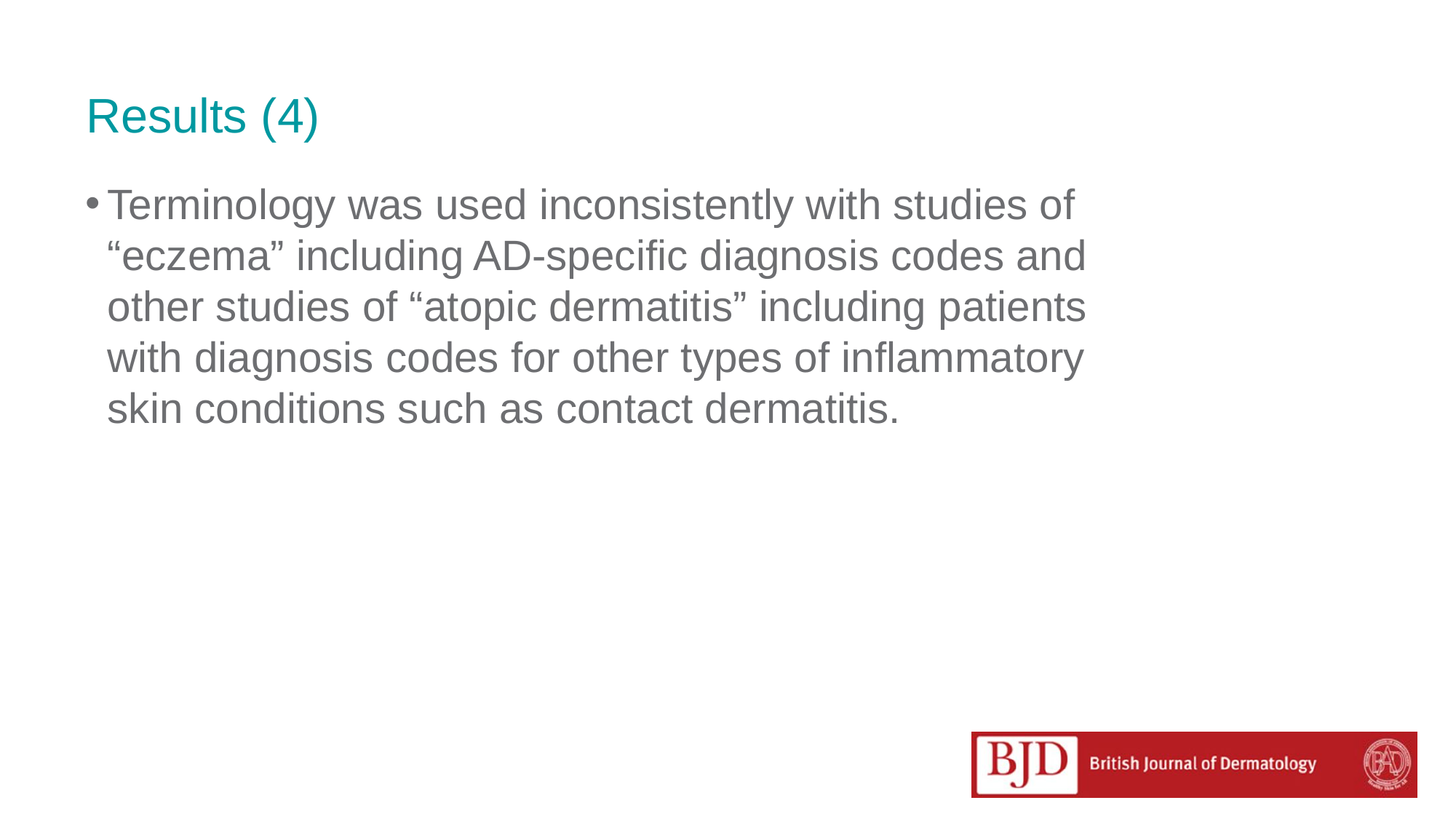

# Results (4)
Terminology was used inconsistently with studies of “eczema” including AD-specific diagnosis codes and other studies of “atopic dermatitis” including patients with diagnosis codes for other types of inflammatory skin conditions such as contact dermatitis.

## Slide 9
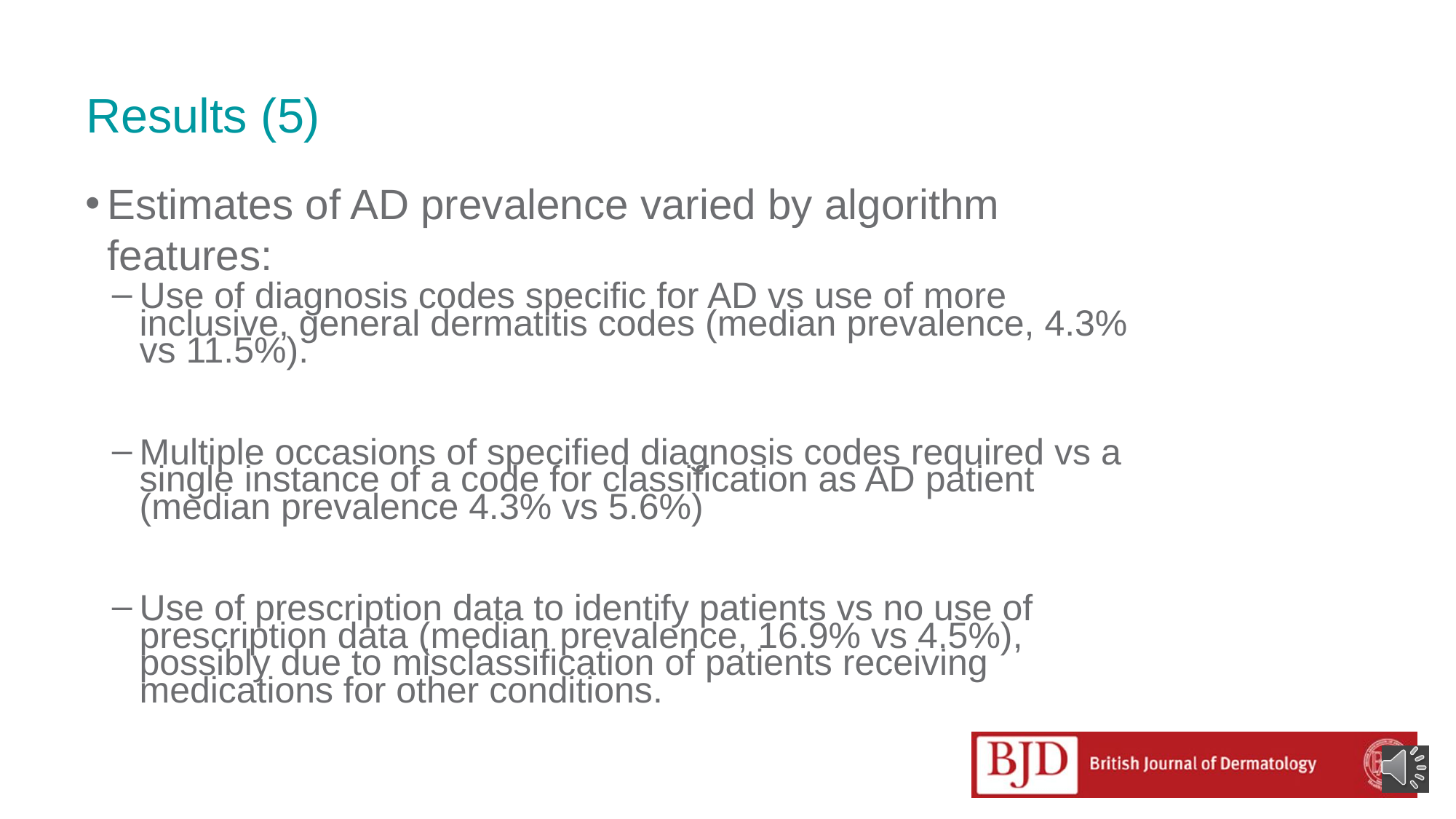

# Results (5)
Estimates of AD prevalence varied by algorithm features:
Use of diagnosis codes specific for AD vs use of more inclusive, general dermatitis codes (median prevalence, 4.3% vs 11.5%).
Multiple occasions of specified diagnosis codes required vs a single instance of a code for classification as AD patient (median prevalence 4.3% vs 5.6%)
Use of prescription data to identify patients vs no use of prescription data (median prevalence, 16.9% vs 4.5%), possibly due to misclassification of patients receiving medications for other conditions.

## Slide 10
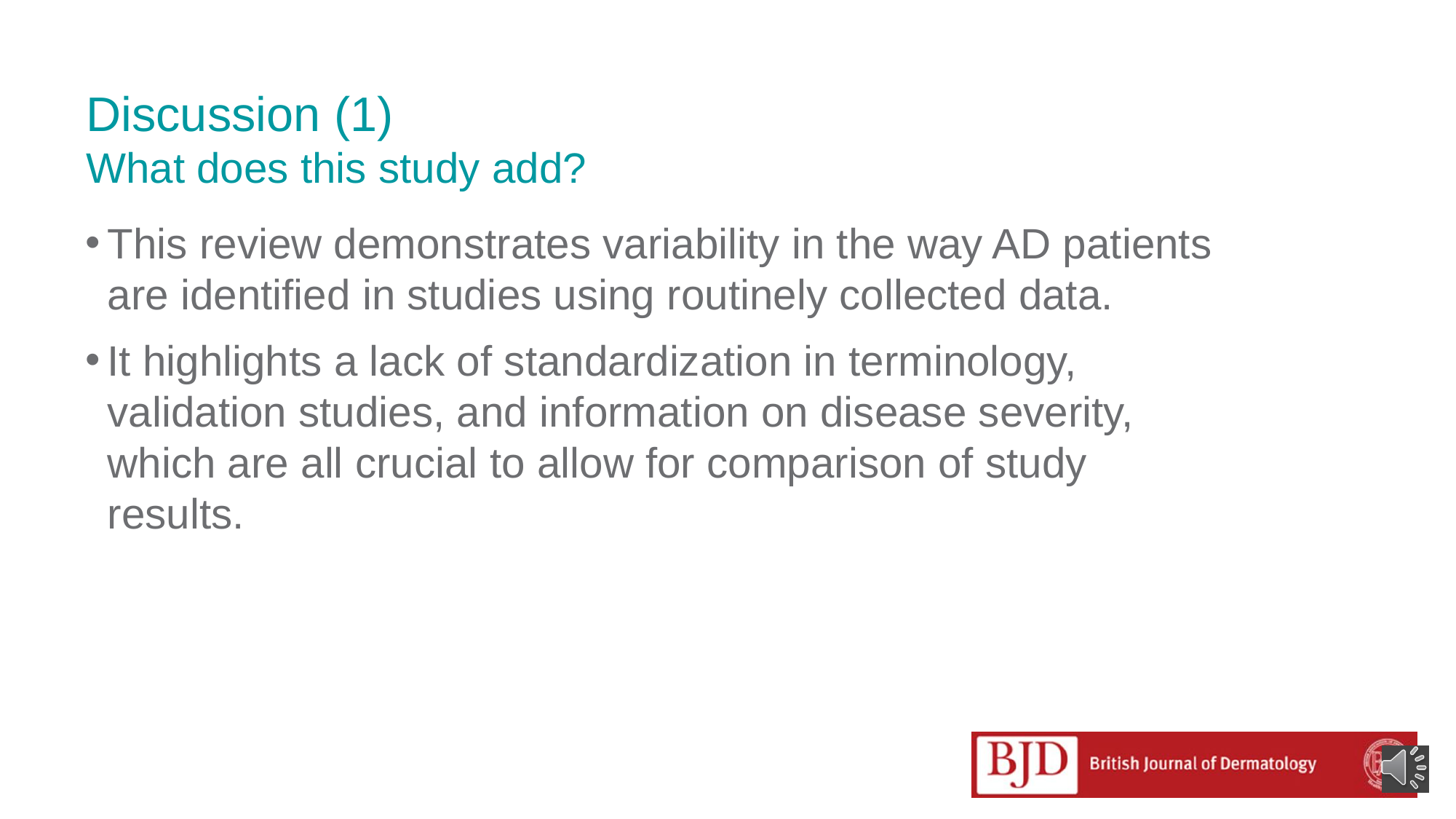

# Discussion (1) What does this study add?
This review demonstrates variability in the way AD patients are identified in studies using routinely collected data.
It highlights a lack of standardization in terminology, validation studies, and information on disease severity, which are all crucial to allow for comparison of study results.

## Slide 11
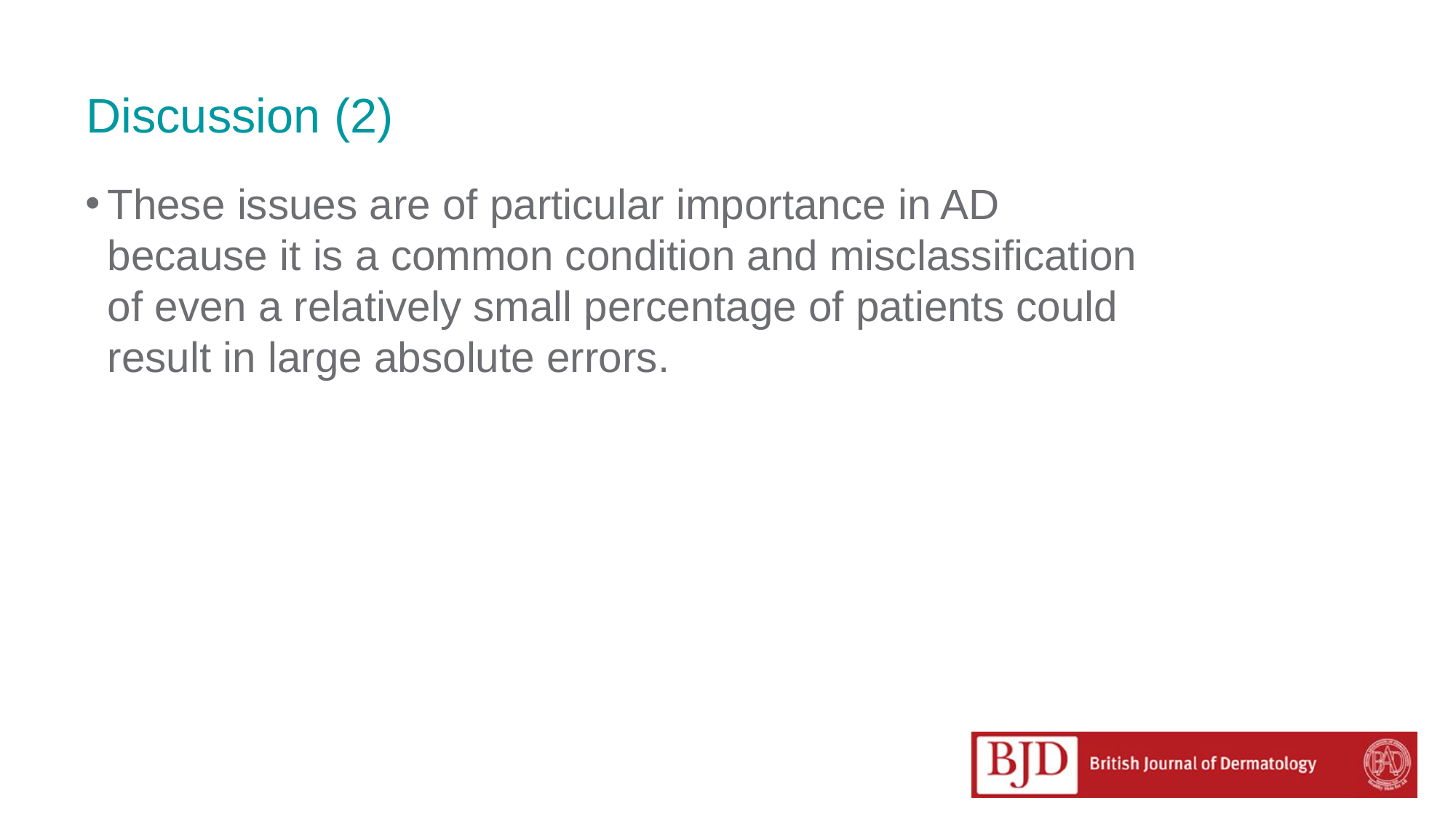

# Discussion (2)
These issues are of particular importance in AD because it is a common condition and misclassification of even a relatively small percentage of patients could result in large absolute errors.

## Slide 12
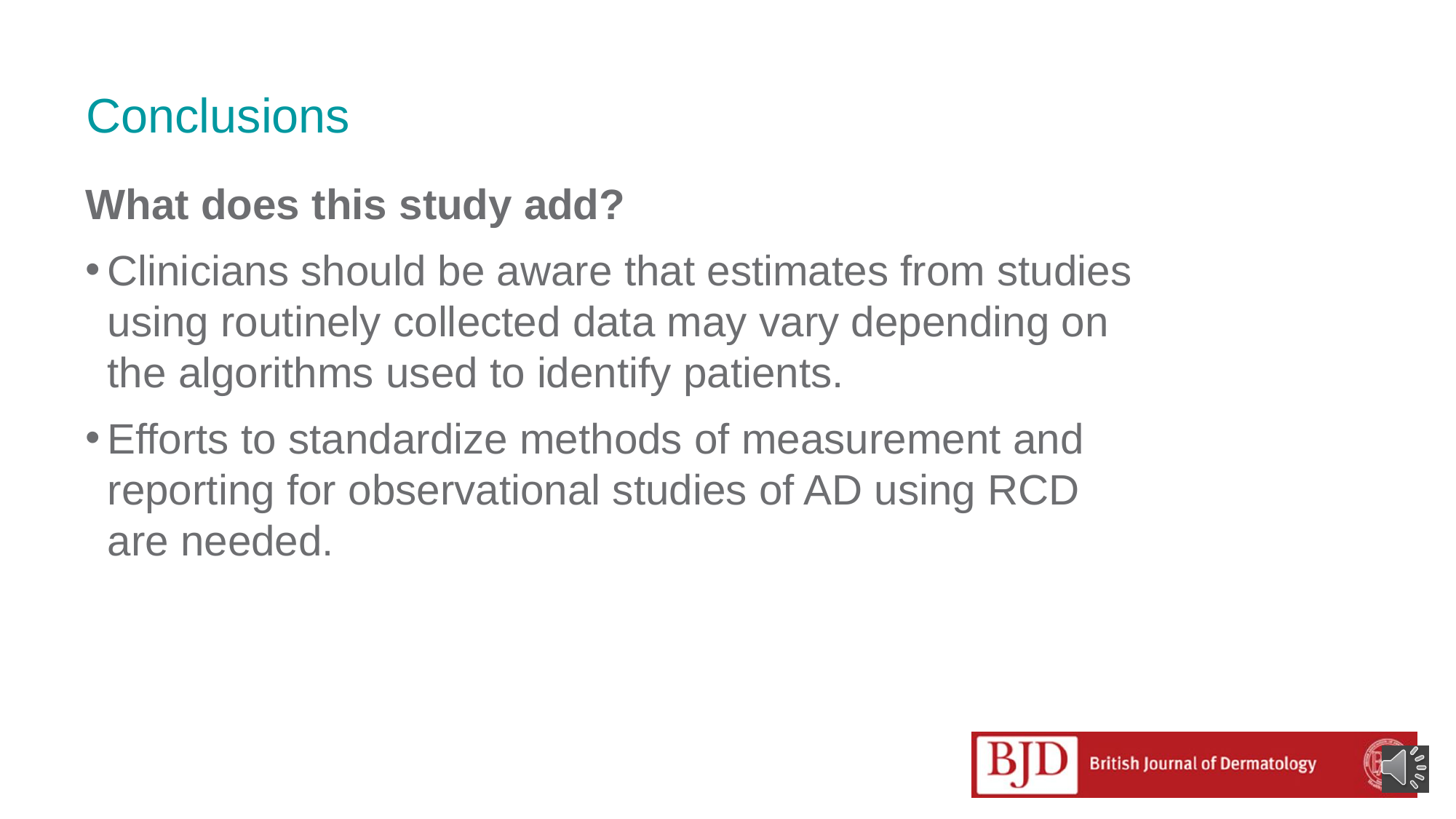

# Conclusions
What does this study add?
Clinicians should be aware that estimates from studies using routinely collected data may vary depending on the algorithms used to identify patients.
Efforts to standardize methods of measurement and reporting for observational studies of AD using RCD are needed.

## Slide 13
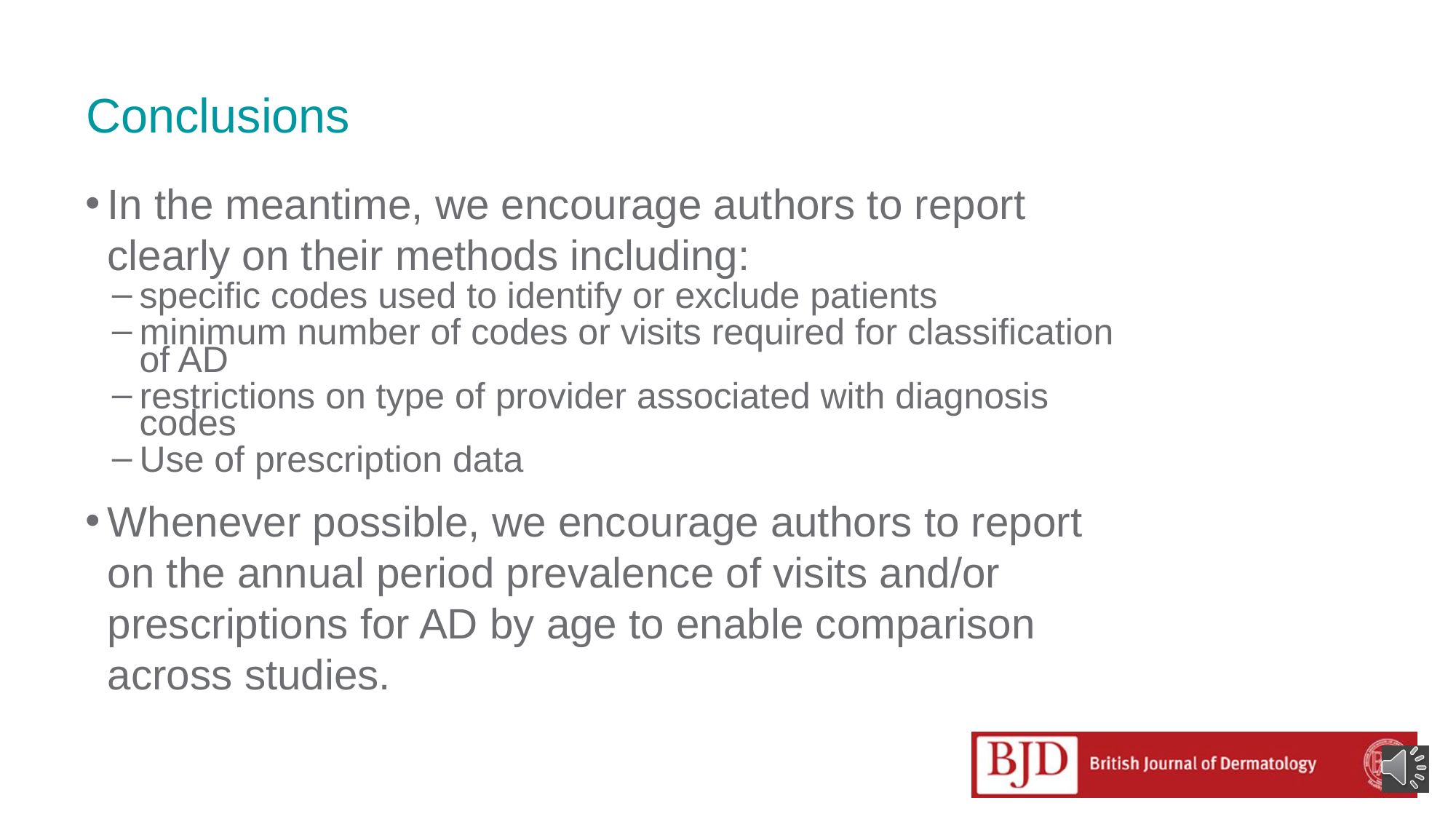

# Conclusions
In the meantime, we encourage authors to report clearly on their methods including:
specific codes used to identify or exclude patients
minimum number of codes or visits required for classification of AD
restrictions on type of provider associated with diagnosis codes
Use of prescription data
Whenever possible, we encourage authors to report on the annual period prevalence of visits and/or prescriptions for AD by age to enable comparison across studies.

## Slide 14
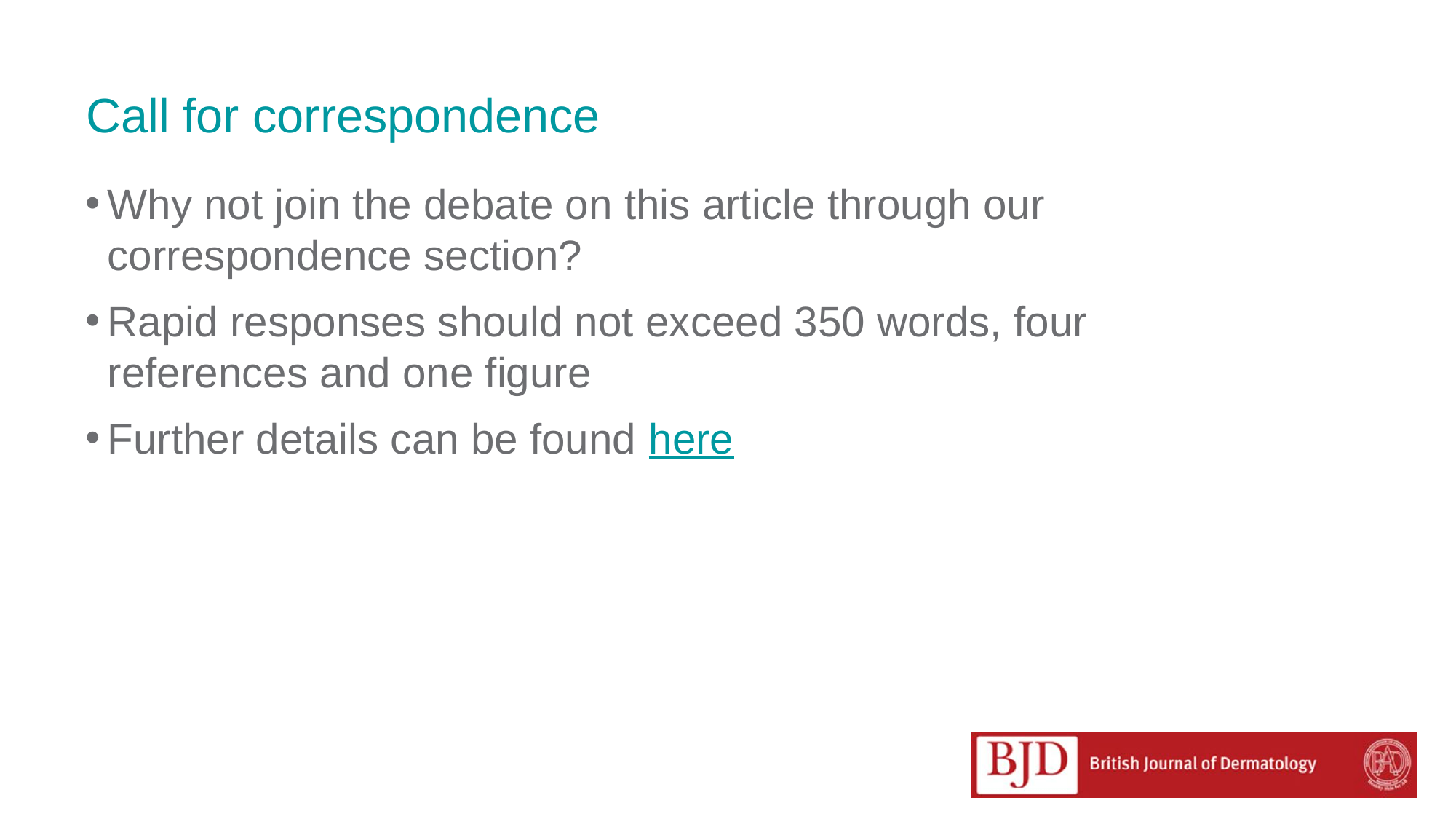

# Call for correspondence
Why not join the debate on this article through our correspondence section?
Rapid responses should not exceed 350 words, four references and one figure
Further details can be found here
